# Mechanical and Impact Damage Analysis on Carbon/Natural Fibers Hybrid Composites: A Review

**DOI:** 10.3390/ma12030517

**Published:** 2019-02-09

**Authors:** Carlo Santulli

**Affiliations:** School of Architecture and Design, Viale della Rimembranza, 63100 Ascoli Piceno, Italy; carlo.santulli@unicam.it

**Keywords:** carbon fibers, natural fibers, impact performance, composites

## Abstract

Hybrid composite laminates including carbon fibers and natural fibers, hence basalt and/or vegetable ones, draw on the experiences accumulated in studying the hybridization of fiberglass with carbon or natural fibers. Yet, in the case of carbon/natural fiber composites, the sense is different: in particular, the idea is to accept the reduction of properties from bare carbon fiber composites and the unavoidable complication in processing, induced by hybridization. The compensation obtained, which offers a rationale to this operation, is the improved toughness and a significant modification of the different modes of failure. This would bring a higher energy absorption and a substantially more effective damage tolerance. The aforementioned characteristics are particularly of interest in the case of flexural properties, impact properties, and residual post-impact performance.

## 1. Introduction

Composite laminates containing more than one type of fiber are defined as “hybrid composites” [1]. The combination of two fibers can be either obtained by stacking layers containing either of the two or intermingling or braiding them across the composite section. The three most diffuse fibers in use for composites are carbon, glass, and aramidic, i.e., Kevlar. Types of hybrids with all the possible combinations among these fibers have been experimented during last decades, up to recent years, namely carbon/glass [2], carbon/Kevlar [3], and glass/Kevlar [4].

In most cases, the hybrid composites that are produced are, for the sake of simplicity in manufacturing, interply hybrids. Here, layers of the two (or more) homogeneous reinforcements are stacked. Other procedures are though possible, such as the fabrication of intraply hybrids (two fiber types in the same layer), intermingled fibers (random mixing), selective placement (a definite fiber is where it is needed for better force), and super-hybrids (placement of each fiber according to a definite orientation) of each fiber composites [5].

The objective in adopting this procedure, which obviously introduces some complications in composite fabrication, is obtaining a positive hybridization effect. This means that the properties obtained are superior to those that would be obtained considering the respective amounts of the originating composites, according to the linear relation that is defined as the “law of mixtures” [6]. In particular, the combined introduction of fibers with distinctly different properties would possibly allow for the tailoring of the final performance of the composites on the requirements from service. The law of mixtures is quite a generalized application, when one refers to static mechanical properties, such as tensile and flexural characteristics, of the composite. In contrast, it may be cumbersome and not a necessarily effective application for dynamic properties, such as impact resistance, which are, on the other side, very important in composites. If this is the case, the properties may be very variable, according to the geometrical patterns selected for hybridization.

The fabrication of hybrids including natural fibers, either of mineral origin, such as basalt, which is directly extracted from a mixture of molten basaltic rocks [7] or extracted from plants, such as hemp, jute, flax, sisal, kenaf, etc., can be recommended in composite materials for different reasons. In particular, concentrating on hybrids including carbon fibers, the hybridization of carbon fiber composites with basalt and with vegetable fibers does come with very distinct characteristics. More specifically, the hybridization of carbon fiber composites with basalt fiber layers does not result in weight gain, although it is expected that the hybrid would present improved impact properties. Conversely, in the case of hybridization with vegetable fibers, some impact resistance penalty is expected, while some weight gain is achieved. For this reason and especially the very high strength and stiffness of carbon fibers, their partial replacement with vegetable fibers remains questionable and has received less attention than is the case for glass fibers. In fact, glass/vegetable fiber hybrid composites benefited during the last decades of a considerable amount of literature. In particular, they were often perceived as a possible replacement for fiberglass, advisable for environmental advantages and for the possible use of sustainable not oil-based matrices [8].

A number of factors have an influence on the properties of the hybrid obtained with respect to the originating composites. The first one of course is the basic physical and mechanical properties of the different fibers; some indications in this sense are offered in Table 1. A few references are also given, bearing in mind that the intervals that are given represent a larger corpus of literature, since, especially as regards carbon and glass fibers, these have been widely investigated. It is notable that, with regards to vegetable fibers, their diameter, beyond being considerably higher than that of monofilament carbon and basalt fibers, can present significant deviations from the average due to the fact that they are formed by a variable number of filaments which are wound together by torsion forces. There are studies that also investigated the decrease in tensile stiffness, which is predictable with an increase in fiber diameter, such as for example in the case of flax [9]. 

**Figure 1:**
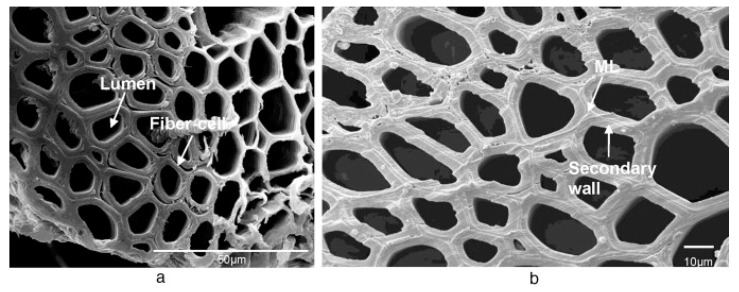
Two views at different magnifications ((**a**): 1000×; (**b**): 2500×) of the sisal fiber cross-section [10]. (From open access publication).

The values in Table 1 report fibers most typically used in composites. These data are also supposed to suggest that for vegetable fibers these data can only be given as a general indication due to their inherent variability owed to species, origin, etc. There is a large amount of literature on the assessment of the tensile properties of vegetable fibers, which presents very variable data. With regards to the measurement of Young’s modulus, it needs to be noticed that the real section of the fiber needs to take into account the presence of lumens, i.e., internal voids, which can take various and not easily predictable geometries. As an example, in Figure 1, lumens of sisal fibers are represented [10]. This means that the real section can be considerably smaller than the one barely measured from diameter. This can be accounted for by using correction factors once, for example using microscopy observations, the percent of internal voids is known. As a matter of fact though, it is evident that introducing the vegetable fiber in a composite with carbon fibers to form a hybrid laminate, strength and stiffness are going to be reduced. In contrast, advantages can come possibly, other than from reduced cost and weight, from modifying the mode of fracture, therefore mitigating the inherent brittleness of carbon fibers.

In terms of costs, to give indications about the possible economical convenience of hybrid production, the approximate prices for plain textile structures with the different fibers are offered in Table 2, bearing in mind that they may vary according to the areal weight, the weave structure, etc.

Another consideration that received less attention but is important nonetheless is the stacking sequence in which the different layers are disposed in the hybrid. In practical terms, there are two general possibilities. The first is inserting the layers reinforced with one of the two fibers in the central section and stacking those with the other fiber on the outer ones, an arrangement often described as the “sandwich hybrid composite”, while the other would allow for layering the fibers reinforced by each of the two in more complex ways, which may be defined as the “intercalated hybrid composite” [16].

The stacking sequence has a particular influence on two correlated characteristics, the damping of the composite [17] and the falling weight impact resistance [18].

The increasing need to mitigate the environmental impact of synthetic fibers and polymers is promoting the use and application of natural materials and orienting the research towards the development of biodegradable systems. This is potentially another reason to produce hybrid laminates including carbon fibers and vegetable fibers, although in practical terms the fabrication of interply hybrids, though simple, does normally exclude fabrication methods to be applied with thermoplastic composites, except possibly vacuum molding.

## 2. Using Carbon Fibers in Combination with Others in Composites

The superior qualities of carbon fibers with regards to tensile strength and stiffness are limited though by some brittleness especially in particular situations, as for example hosting junction elements, such as bolts and rivets, and therefore needing to be machined for the purpose. In this case, it proved suitable to obtain more ductile fracture hybridization with other fibers, the first attempts being with glass ones [19]. Concerns were raised with regards to the drop of the fracture energy of the notched glass/carbon hybrid laminates undergoing Charpy impact testing as far as the ratio between carbon fibers and glass fibers was increased [20].

In practice, passing to falling weight impact damage, an effect of improving strain to failure brought to higher impact resistance was obtained by hybridizing the carbon fiber composites with glass fiber reinforced layers, although in practical terms the result highly depended on the stacking sequence adopted [21]. Concentrating on the impact resistance for energies as high as 40 Joules, the improvement was so large, the coupling plain weave glass fiber laminates with twill weave carbon fiber laminates had to compensate for the slight reduction in stiffness [22]. On the other side, with regards particularly to the load bearing components for the automotive industry, fiberglass was hybridized with carbon fiber laminates to reduce their weight. Static tests indicated that hybrid composites with 50/50 carbon and glass fiber reinforcement proved effective in flexure with outer carbon layers, whereas an intercalated structure offered the highest compressive strength, with tensile tests basically unchanged with whichever of the two dispositions was adopted [23]. This has been questioned in other works, which suggested that the hybrid composites with glass fabric layers in the exterior and carbon fabric layers in the interior offered higher tensile strength and ultimate tensile strain than the reverse [24]. A possible interpretation of this result is that the influence of the different weave structures has to be taken into account (in this specific case, satin for carbon fiber and plain weave for glass).

To summarize these general considerations, it can be suggested that the possible factors influencing the properties of hybrid composites including carbon fibers are either connected to the composite structure (weave structure, stacking sequence, and amount of reinforcement introduced) or to the production (matrix employed or manufacturing method used). In practical terms, as in the next section, the possibilities applied appear quite limited so far, which are described in more detail.

## 3. Studies on Hybrid Composites Including Carbon and Natural Fibers

Some comprehensive reviews about hybridization in polymer composites [25] or about the more concentrated particular aspects, which are critical for their application, such as impact [26], do exist already. In practical terms, the rationale of fabricating a hybrid including carbon fibers with natural fibers would principally be the possibility of obtaining different modes of damage propagation, possibly less “abrupt”, to adapt to possible applications. This would occur even with some slight penalties in terms of tensile stiffness or strength with respect to carbon fiber composites.

**Figure 2:**
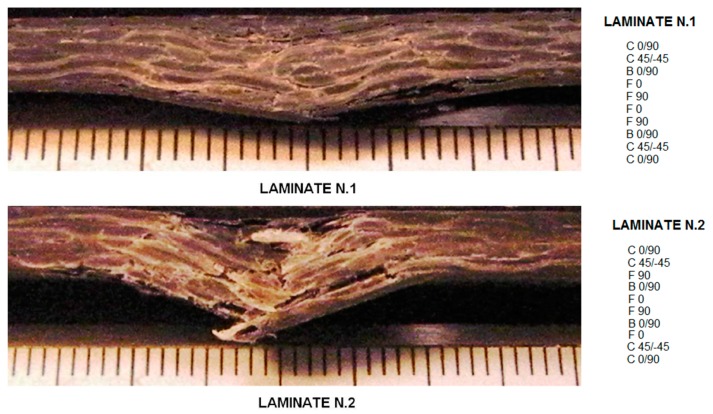
The impact damage at 38.4 Joules on two different configurations of carbon/basalt/flax fiber hybrid laminate [27]. (Reproduction permission obtained).

More in particular, hybrids including carbon and natural fibers can be divided in two categories: one that is realized using basalt fibers and the other that includes other fibers, namely vegetable ones. A single work, [27], was found, which was based on more complex hybrids, including, at the same time, carbon, basalt, and flax in two different configurations. The study was concentrated on the falling weight impact performance keeping carbon fiber layers as the external ones, though ideally creating a softer core including basalt and flax fiber layers with the idea to provide improved energy dissipation. Here, two configurations were compared, and it was suggested that concentrating the flax fiber reinforced layers at the geometrical center of the laminate, such as in laminate n.1 in Figure 2, resulted in some kind of “locking effect” during the damage, which effectively contributed to damage dissipation.

Studies that are currently available on this topic are reported in Table 3. It is noteworthy that most work has been performed using epoxy resins as the composite matrix, since composite manufacturing to obtain hybrids is normally carried out completely at ambient temperatures. In principle, epoxy is not the most compatible matrix for use with natural fibers, in particular with the ones of vegetable origin, such as flax, hemp, etc. On the other side, introducing plant fibers into carbon fiber composites gives to these also some hydrophilic character, of course more pronounced the higher the amount of plant fibers used. Using epoxy reduces the possible effect of this hydrophilic character on the performance of the composite when exposed to harsh environmental conditions (e.g., seawater). It is also important to take into account the fact that carbon fiber composites have a typical market for long life and high-performance applications, e.g., in nautical, aeronautical, and sport automotive industries. In this particular scenario, the use of more resistant thermosetting matrices, such as epoxy, is often recommendable. In order to reduce the environmental impact in the production of epoxy, it is worth noting that in recent years, the use of bio-epoxy, originating for example from cardanol, which has been experimented with for application in natural fiber composites or for example with flax [28] or hemp [29], did not prove popular on carbon fiber composites so far and also because the sustainability rationale is limited in that case.

The discussion around the aforementioned studies needs to be concentrated on the rationale for introducing further complications in fabricating hybrid laminates starting from carbon fiber reinforced composites. The reason can be different yet it is mostly concentrated about the need to obtain a more ductile behavior from carbon fiber composites, though possibly not losing too much in terms of tensile and flexural strength and stiffness. This general purpose is declined in different forms, pertaining to several application fields. This suggests that studies on this kind of hybrid are going to progress further in the future, aiming at attaining the desired properties for application.

**Figure 3:**
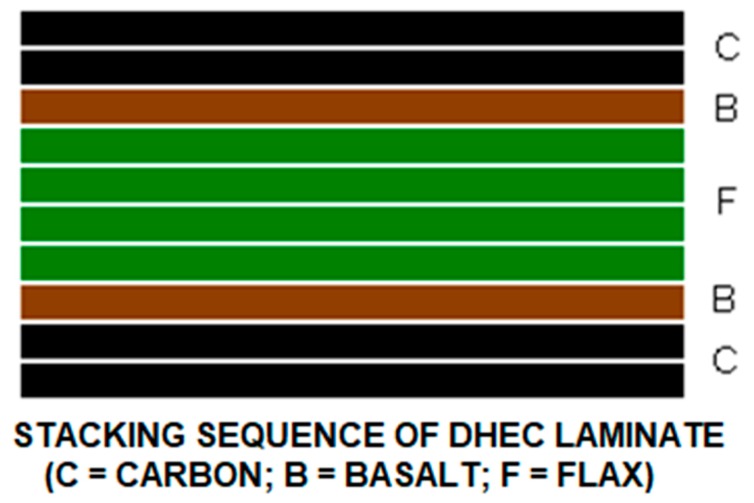
The DHEC laminates (original picture from the author of the chapter) [33].

**Figure 4:**
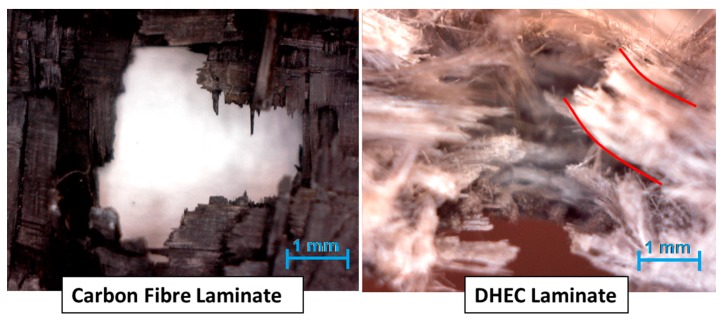
A comparison of the mode of penetration due to hybridization. (Original picture from the author of the chapter).

**Figure 5:**
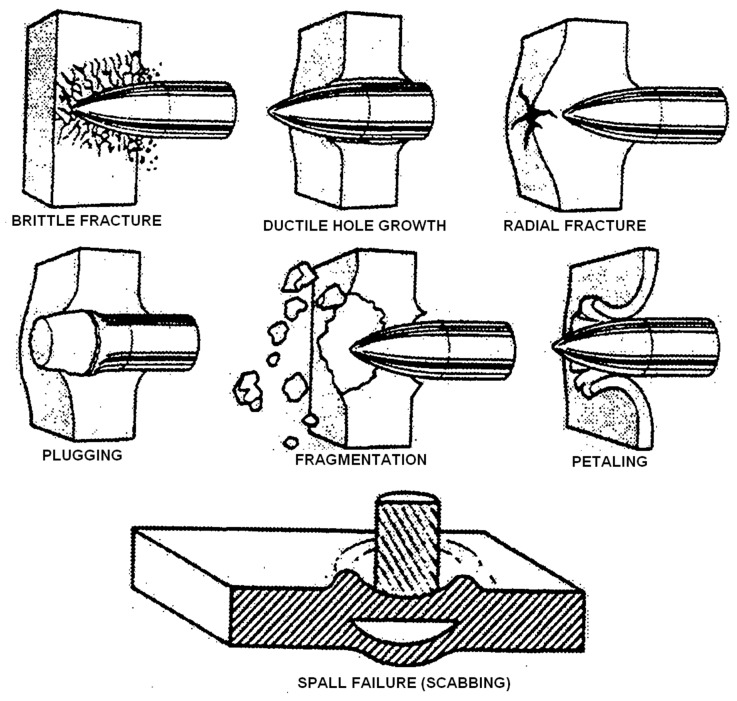
The typical failure modes for a ballistic impact.

In the particular case of work in [33], carbon–flax–basalt hybrids referred to as DHEC (Ductile High Energy Composites) were developed and studied, which the stacking sequence of has been reported in Figure 3. The aim of their production was in particular to offer a more advantageous performance, resulting in improved energy absorption during ballistic impact. In particular, the mode of fracture was considerably different, as suggested from the indications of the perforation mode, compared to that of a carbon fiber composite of equal thickness, which appeared considerably less “abrupt” and with geometrical complexity (Figure 4). This indicated the occurrence of a more effective energy dissipation process during the time that was required for penetration. While the carbon fibers are interrupted due to shear forces leading to filaments breaking with no noticeable deformation, in the case of the DHEC composite, a “petaling” behavior is observed due to the “crater shaped” and highly deformed section of the hole. It needs to be observed that petaling and plugging are a predominant ballistic perforation mode for ductile thin plates and they are typically offered from fiberglass [42] (Figure 5) yet not from carbon fiber composites: hybridization with basalt and flax allowed obtaining this though.

**Figure 6:**
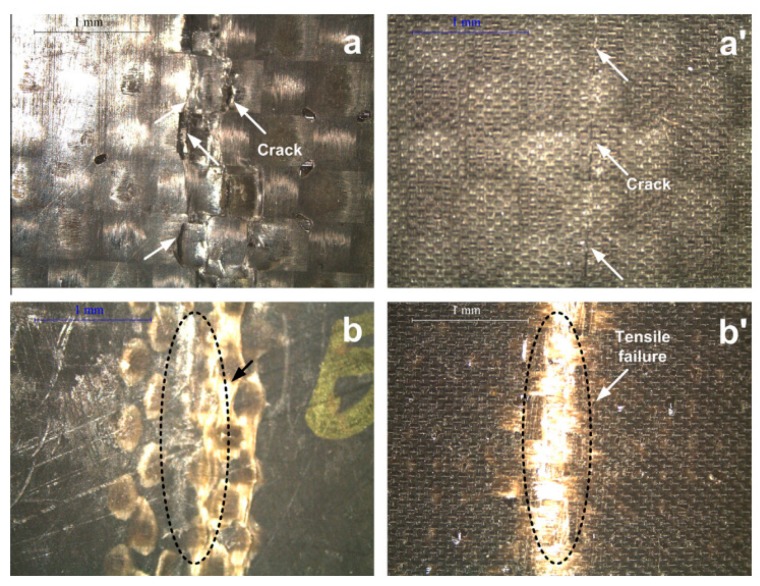
The failure surfaces of hybrid laminates after a flexural test: carbon fabric ((**a**) and (**a’**)) and basalt fabric ((**b**) and (**b’**)) at the compressive side (**a**,**b**) and at the tensile side (**a’**,**b’**), respectively (original picture from the author of the chapter).

Studies in [31,32] investigated in particular the effect of the stacking sequence adopted on the properties of carbon/basalt fiber composites. In particular, passing from pure carbon fiber composite to pure basalt fiber one, the flexural strength is reduced by slightly over 50%: the values for the hybrids are in between, and considerable differences among stacking sequences are observed. A different behavior is reported in Figure 6: both at the compressive and tensile side, the brittle behavior of carbon fibers can be recognizable with respect to that of basalt fibers. These considerations are based on the underlying fact that flexural properties are controlled by those of the outermost layer. In particular, a number of carbon (C)/basalt (B) hybrids were fabricated in configurations such as CBC and BCB with a variable number of layers of each composite. This allowed a quite accurate modeling of the properties, which was possible up to a deviation of around 5% from the predicted values.

**Figure 7:**
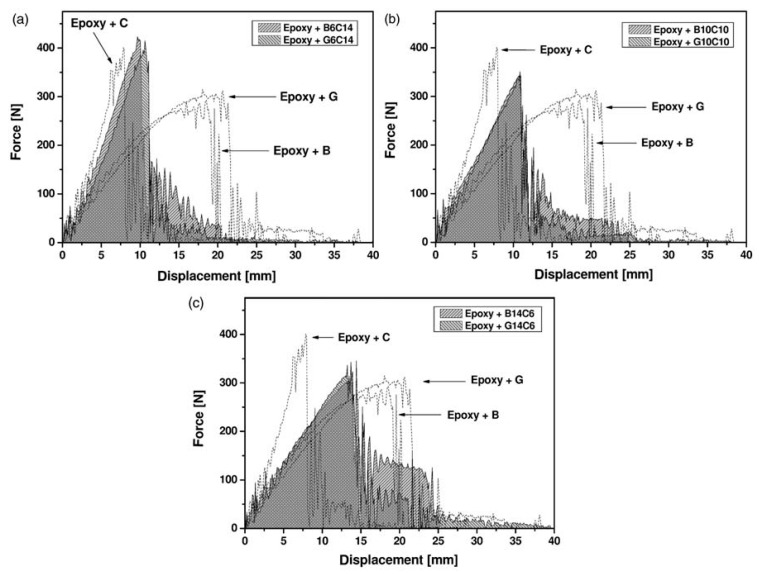
The Charpy impact tests curves of glass/basalt and carbon/basalt hybrid laminates with respect to the originating laminates: (**a**) Laminates with 14 carbon fiber layers and 6 layers of another fiber (glass or basalt) (**b**) Laminates with 10 carbon fiber layers and 10 layers of another fiber (glass or basalt); (**c**) Laminates with 6 carbon fiber layers and 14 layers of other fiber (glass or basalt) [30]. (Reproduced from copy available at www.unitn.it).

The work in [30] concentrated on the effect of carbon/basalt hybridization on interlaminar shear strength (ILSS) (Table 4) and Charpy impact properties (Figure 7). They found out that the introduction of basalt fibers in carbon fiber laminates could promote an increase of the adsorbed impact energy, enhancing the capability of laminates to sustain damage propagation and delamination without catastrophic failure. This was despite the fact that both ILSS and Charpy impact properties indicated a reduction of the absorbed energy with respect to the originating laminates. As a matter of fact though, this suggests that for this kind of test, a model using simple equations in the rule-of-mixtures style cannot reasonably be proposed. This is only possibly applicable to tensile and flexural tests where the mode of fracture does not substantially change with hybridization.

**Figure 8:**
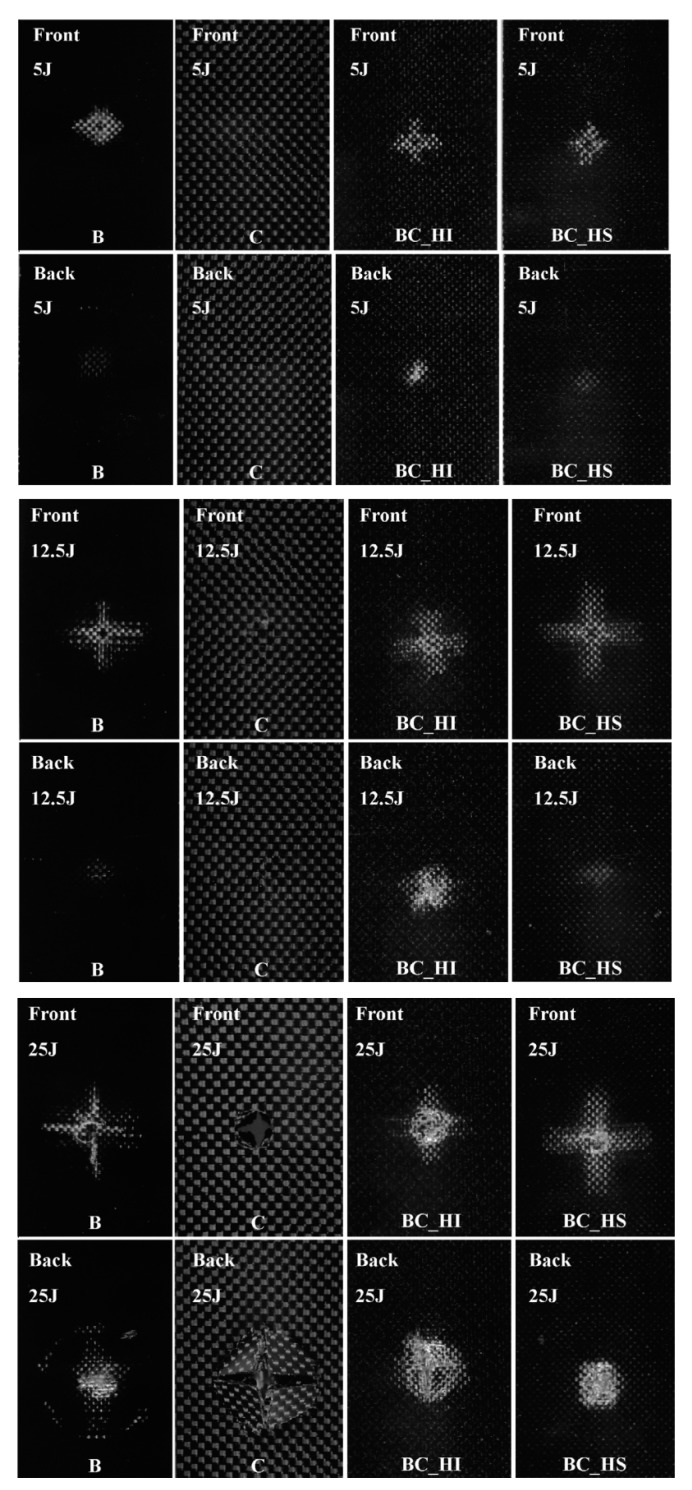
The sandwich and intercalated carbon–basalt hybrid laminates compared with pure carbon and pure basalt laminates at different impact energies (from the original supplied by the authors of [18]).

**Figure 9:**
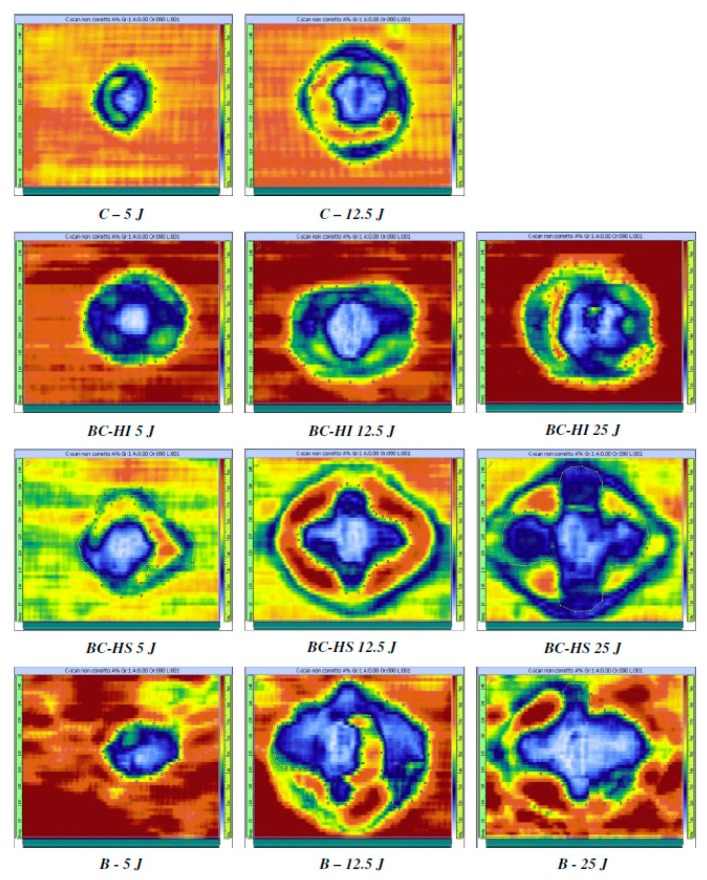
The C-Scan images of the impacted sandwich and intercalated carbon–basalt hybrid laminates compared with the pure carbon and pure basalt laminates at different energies (redrawn from an image supplied by authors of [18]).

To see the global effect on the energy absorption of the adoption of different stacking sequences, namely sandwich-like (S) and intercalated (I), for carbon/basalt hybrid laminates, the falling weight impact and post-impact residual flexural tests were performed in [18]. Results indicated that the intercalated configuration (alternating sequence of basalt and carbon fabrics) offered a better impact energy absorption capability with respect to the all-carbon laminates (Figure 8). This was due to the possibility to better contain the damage in a restricted area, as reported also from C-Scan monitoring in Figure 9. On the other side, sandwich-like configuration 3B-7C-3B presented the most favorable flexural behavior.

**Figure 10:**
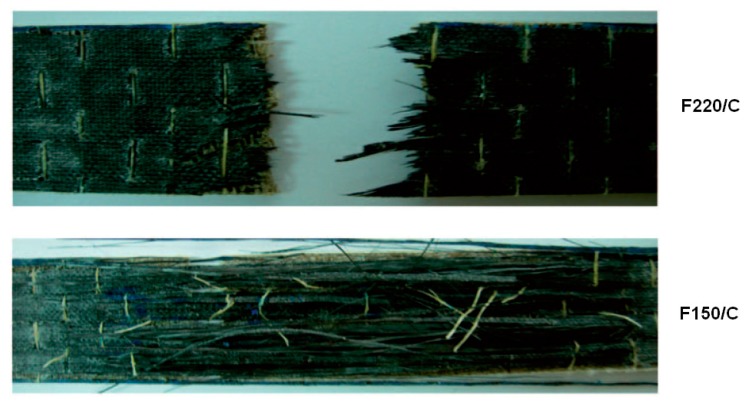
The tensile fracture of the carbon/flax hybrid laminates.

Most studies on carbon fiber hybrids with vegetable fibers concerned the use of flax: in every case, modeling their behavior appeared to be a significant concern. Despite this, the application of a rule of hybrid mixtures (ROHM) from data obtained from the vibration modes proved effective in the prediction of tensile data; in contrast, the flexural ones demonstrated to be lower than expected [34]. However, this conclusion did not prove to be of general application: in particular, the influence of the type of weaving structure adopted appeared to have a significant importance. As an example of this difficulty, in [37] two different bidirectional flax fabrics, with respective areal weight equal to 150 and 220 g/m², were used in combination with a unidirectional ultra-high modulus (UHM) carbon fabric: the latter was placed on the outermost part of the laminate. While the flexural properties of the flax fiber laminates including the 150 g/m² fabric were higher, they were in contrast to the tensile properties of that including the 220 g/m² one. Simple prediction of the properties of the hybrid with respect to the originating laminates, using ROHM, was demonstrated to be very inaccurate, as reported in Table 5, particularly for tensile performance. This was attributed to the different modes of tensile failure of the laminates, which was based on the fiber fracture in 220 F/C while involved the delamination in 150 F/C, as depicted in Figure 10.

**Figure 11:**
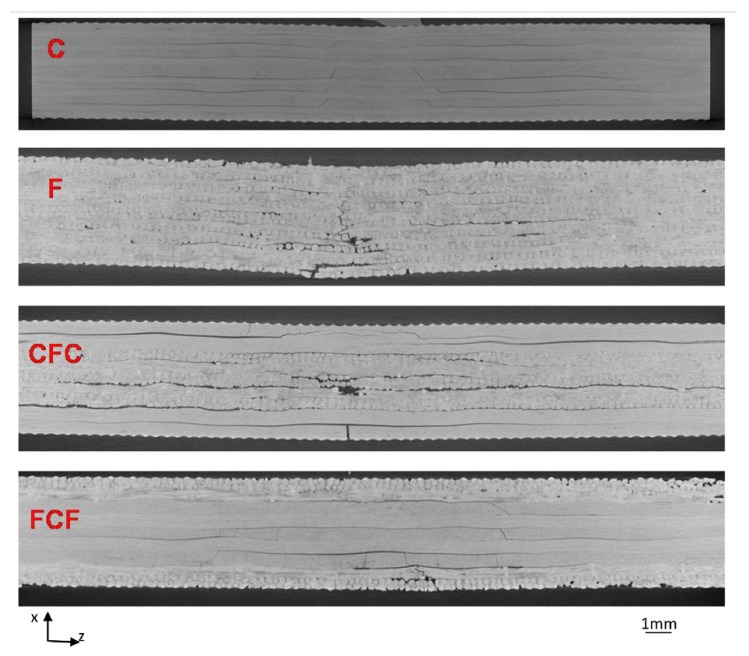
The computed tomography of different flax, carbon, and flax/carbon hybrid laminates impacted at 10 Joules [36] (Reproduction permission obtained).

The prediction of the hybrid performance from that of the originating laminates proved even more difficult in the case of dynamic performance, such as it is the case for impact testing. Falling weight impact tests have been performed in [36], on two different carbon flax configurations, reported in Table 6, proposing also the introduction of flax on the external surfaces of the hybrid, with the idea that this could result in some more energy dissipation at a low impact. This was in effect encountered, for example, for impact at 10 Joules, although on the other side, a pure carbon fiber laminate provided no evidence of damage up to energies higher than this (Figure 11).

A concern about the application of vegetable fibers, such as flax, in hybrid composites is that a hydrophilic component is introduced in a fully hydrophobic context, which means that some water absorption becomes possible. Work carried out in [35] demonstrated that carbon fiber laminate hybridization with unidirectional flax pre-pregs led to a much lower water absorption than it was the case for cross-ply flax ones, 2 wt % against 8 wt % after immersion for 27 days. On the other side, the adaptability and toughness of flax fibers would allow the possible use of carbon/flax hybrids in fields, such as in orthopedic bone plate replacements, to limit the consequences of catastrophic brittle failure of the carbon/epoxy laminates. This was attempted in [38], although the right balance between the amount of flax and carbon fiber to be introduced in the composites proved not obvious to establish.

Other than flax, other vegetable fibers have been proposed for hybridization with carbon fibers, in particular the hybridization with hemp, a plant which provides fibers that are particularly of interest as they offer thermal insulation properties [43], was investigated by Scutaru and Baba, 2014, in the view of offering more information about low speed impact as a simulation for accidental dropping events on a composite panel. Further, early studies investigated further fibers for possible hybridization with carbon fibers in composites, providing information on the chemical resistance of sisal [40] and suggesting the application of kenaf in a thermoplastic rubber matrix, unusual for hybrid laminates production [41], respectively. The former study suggested significant reasons for the concern about the chemical resistance of sisal against carbon tetrachloride, while the latter, being based on a short kenaf fiber introduction, gave promising evidence for flexural results, only up to 15 vol.% of overall fiber content.

As a general consideration, the hybridization of carbon fiber laminates with natural fibers, even though it may provide reduction of costs, is limited so far to cases in which different modes of energy dissipation are desired and/or it is desirable to reduce the brittleness of the composite. This is achieved in particular by modifying the mode of failure of the laminate by introducing other fibers, although the preventive measurements of the properties of the hybrid from those of the originating laminates may not always be obvious. However, it is suggested that this is considered in future works, especially in fields involving impact damage by accidental events or ballistic application.

## 4. Conclusions

The production of hybrid composite laminates including carbon fibers and natural fibers, hence basalt and/or vegetable ones, involves particular complications, which limit their fabrication so far mostly to the manufacture of interply hybrids and to using thermosetting matrices. However, this may have some significance in particular situations, such as the attenuation of brittleness and abrupt failure, such as at low speed impact or ballistic impact. The behavior of these hybrids appears to be possibly modeled in cases such as tensile and flexural testing yet not to where the effect of interlaminar adhesion becomes predominant, such as it is usually the case for interlaminar shear strength or impact strength. In all cases, the adoption of different stacking sequences does widely affect the results obtained, and it needs to be considered that the use of vegetable fibers, though might have some environmental merits, on the other side introduces problems, such as the dimensional variability of reinforcement and the sensitivity to water absorption.

## Figures and Tables

**Table 1:** Some properties of carbon, basalt, and some vegetable fibers.

Fiber	Diameter (µm)	Tensile Strength (MPa)	Tensile Modulus (GPa)	Density (g/cm³)	References
Carbon	5–10	2000–5000	200–600	1.8	[11]
E-glass	7–25	1950–3500	70–80	2.55	[12]
S-glass	8–12	4500–4700	75–90	2.5	[13]
Kevlar	12	3000–3150	63–67	1.4	[13]
Basalt	10–20	2800–3100	80–90	2.6–2.7	[11]
Flax	12–20	400–600	12–25	1.2–1.5	[11]
Hemp	25–500	300–700	20–70	1.3–1.5	[14]
Sisal	11–22	350–700	7–22	1.4–1.5	[15]
Kenaf	30–40	150–250	10–20	1.1–1.2	[11]
Jute	17–20	350–780	20–30	1.3	[8]
Coir	10–24	550–650	4–6	1.2	[13]

**Table 2:** The approximate price of fiber textile products.

Fiber Textiles (Plain Weave)	Approximate Price ($/kg) (January 2019, Elaborated from Alibaba)
Carbon	35–60
E-glass	1–2
S-glass	3–7
Kevlar	50–150
Basalt	20–70
Flax	12–20
Hemp	5–13
Kenaf	1–3
Jute	0.50–1.50
Sisal	1.50–2.50
Coir	3–8

**Table 3:** Studies on hybrid composites including carbon fiber laminates.

Fibers other than Carbon	Matrix	Evaluations	References	Main Conclusions
Basalt	Epoxy	Interlaminar shear strength, Charpy impact strength	[30]	Flexural moduli of the hybrid composites followed the rule of mixtures. Ultimate properties and Charpy impact tests revealed positive deviations and therefore synergistic effects, due to the presence of both carbon and basalt fibers. The introduction of basalt fibers in the carbon fiber laminates promoted an increase of the adsorbed impact energy, avoiding catastrophic failure.
Basalt	Epoxy	Low speed impactFour-point flexural (pre- and post-impact)	[18]	In impact loading, the basalt fiber hybridization enhances peak force while preventing penetration. This is due to the higher ductility of basalt fibers that results in a wider damaged area and more energy absorption. Sandwich laminates are superior in terms of static properties (flexural and interlaminar shear yet more sensitive than intercalated hybrids to the effect of impact damage.
Basalt	Epoxy	Flexural	[31]	Application of the hybrid mixture law led to observing that differences between the calculated and experimental values for the flexural properties were less than 5% for all sandwich hybrids.
Basalt	Epoxy	Flexural	[32]	Higher flexural strength and stiffness were obtained by stacking carbon fiber layer at the compressive side. The best performance was obtained by sandwiching foru basalt fiber layers between three carbon fiber layers on each side
Basalt and flax	Epoxy	Tensile, flexural, interlaminar shear strength test, falling weight impact	[27]	Differences in terms of mechanical and impact performance between selecting an either sandwich or intercalated performance for the laminates, keeping basalt fibers on the outer surfaces were in general terms quite limited. Of course, intercalation increases the complexity of manufacturing, which suggests their application should be limited to specific cases, for example for the need to better disperse damage
Basalt and flax	Polyurea	Tensile, flexural, interlaminar shear strength, high speed impact	[33]	The hybrid with basalt and flax showed a significantly improved mode for energy absorption during ballistic impact with respect to carbon fiber laminates with comparable properties (hence lower thickness). This was due to the different damage progression and improved ductility.
Flax	Epoxy	Tensile, flexural. damping loss, elastic modulus by vibration	[34]	The application of the rule of hybrid mixtures (ROHM) was proposed for carbon–flax hybrids, obtaining positive deviations for experimental results, except for flexural data
Flax	Epoxy	Damping modeling	[17]	The major role of the position of flax fiber layers into hybrids as for flexural and damping properties was highlighted
Flax	Epoxy	Tensile, flexural, thermal degradation, water absorption	[35]	Comparisons for carbon fiber laminates with two types of flax/carbon hybrids laminates including either cross-ply (CP) or unidirectional (UD) flax fabric indicates that hybrids with UD flax were competitive with carbon fiber laminates as for level of water absorption. The elongation at break was increased by hybridization, though critical behaviors were indicated by higher weight loss in hybrids during thermal degradation
Flax	Epoxy	Four-point flexural, low speed impact	[36]	The work compared carbon/flax hybrid laminates having either flax or carbon on the outer surfaces (FCF or CFC). It was confirmed the superiority of the latter as for flexural performance, whereas with impact with no penetration in the presence of flax on the outside allows for better dispersing damage in the laminate
Flax	Epoxy	Tensile, flexural	[37]	The study was based on the comparison of the effect of two different stacking sequences (150 and 200 g/m^2^) for flax fabric introduction. The former proved superior for tensile and flexural performance of the hybrid.
Flax	Epoxy	Tension, three-point bending, Rockwell hardness	[38]	The hybrids were closer in structure to human cortical bone than orthopedic metal plates, an ultimate tensile strength and modulus of 399.8 MPa and 41.7 GPa, and an ultimate flexural strength and modulus of 510.6 MPa and 57.4 GPa, respectively.
Hemp	Unsaturated polyester	Low speed impact	[39]	Falling weight impact resistance was provided for these laminates (4 mm thickness) up to an impact velocity of 3 m/s
Sisal	Unsaturated polyester	Tensile, flexural, chemical resistance	[40]	Alkali treatment by boiled sodium hydroxide solution improved tensile and flexural properties also in the sisal/carbon fiber with respect to the untreated fiber reinforcement. This was attributed to hemicellulose removal.
Kenaf	Thermoplastic rubber	Flexural, impact, dynamic mechanical analysis	[41]	Flexural strength and stiffness increased up to 15% of global volume of fibers, then declined, while impact strength showed an increase even for 20% global volume of fibers.

**Table 4:** The interlaminar shear strength of glass/basalt and carbon/basalt hybrid laminates with respect to the originating laminates [30] (from copy available at www.unitn.it).

Reinforcement (Total 20 Layers) (Epoxy Matrix)	ILSS (MPa)
G20	59.7 ± 1.4
B20	60. 2 ± 0.9
C20	55.7 ± 2.1
B6C14	45.2 ± 0.9
G6C14	43.5 ± 1.3
B10C10	46.9 ± 0.6
G10C10	45.9 ± 2.0
B14C6	53.2 ± 1.0
G14C6	54.0 ± 0.5

**Table 5:** The (**a**) flexural and (**b**) tensile properties for flax and flax/carbon laminates. (from data supplied from authors of [37]).

**a. Flexural Properties for Flax and Flax/Carbon Laminates**		
**Laminate**	**E (GPa)**	**σ (MPa)**
F150	7.41 ± 0.39	76.42 ± 3.59
F220	5.35 ± 0.21	61.05 ± 2.28
F150/C	23.84 ± 0.74	160.42 ± 10.46
F220/C	14.41 ± 1.38	85.00 ± 5.38
**b. Tensile Properties for Flax and Flax/Carbon Laminates**		
**Laminate**	**E (GPa)**	**σ (MPa)**
F150	1.79 ± 0.04	78.63 ± 1.41
F220	4.5 ± 0.15	90.43 ± 1.47
F150/C	6.48 ± 0.32	288.03 ± 30.23
F220/C	5.09 ± 0.34	172.4 ± 25.5

**Table 6:** The configurations for the carbon–basalt fiber hybrid composites study [36]. (Reproduction permission obtained).

Laminate	Stacking Sequence	Number of Flax Layers	Number of Carbon Layers	Total Fibre Volume Fraction (%)
F	[(0/90)_4_/0]_s_	18	-	56 ± 0.1
C	[(0/90)_3_/0]_s_	-	14	59 ± 0.1
FCF	[(0_2_/90_2_)^F^/[(0_2_/90_2_)^C^/0^C^]_S_	8	10	62 ± 0.1
CFC	[(0_2_/90_2_)^C^/[(0_2_/90_2_)^F^/0^F^]_S_	10	8	60 ± 0.1
